# Adverse Childhood Experiences, Neurocognitive Functions, and Long-Term Mortality Risk

**DOI:** 10.1001/jamanetworkopen.2025.31283

**Published:** 2025-09-10

**Authors:** Jing Yu, Denise L. Haynie, Rajeshwari Sundaram, Stephen E. Gilman

**Affiliations:** 1 Social and Behavioral Sciences Branch, Division of Population Health Research, Eunice Kennedy Shriver National Institute of Child Health and Human Development, Bethesda, Maryland; 2 Biostatistics and Bioinformatics Branch, Division of Population Health Research, Eunice Kennedy Shriver National Institute of Child Health and Human Development, Bethesda, Maryland; 3 Department of Mental Health, Johns Hopkins Bloomberg School of Public Health, Baltimore, Maryland

## Abstract

**Importance:**

Higher intellectual abilities have been associated with lower mortality risk in several longitudinal cohort studies. However, these studies did not fully account for early life contextual factors or test whether the beneficial associations between higher neurocognitive functioning and mortality extend to children exposed to early adversity.

**Objective:**

To explore how the associations of child neurocognition with mortality changed according to the patterns of adversity children experienced.

**Design, Setting, and Participants:**

The Collaborative Perinatal Project (CPP) is a US pregnancy cohort study conducted at 12 US academic medical centers among children born between 1959 and 1966. Pregnant women at participating centers were invited to enroll in the study, and their offspring were followed up for assessments of neurocognition and mortality risk. The CPP offspring cohort was linked to the National Death Index to ascertain their later vital status. Analyses were conducted in August 2024 and June 2025.

**Exposure:**

Nine neurocognitive test scores captured children’s visual-motor, sensory-motor, auditory-vocal, intelligence quotient (IQ), and academic skills.

**Main Outcome and Measure:**

All-cause mortality between 1979 and 2016.

**Results:**

Among the 49 853 CPP offspring, about half were male (25 226 offspring [50.6%]). A total of 23 331 (46.8%) mothers were Black, 3739 (7.5%) were other races, and 22 783 (45.7%) were White Eight of the 9 neurocognitive scores were associated with the risk of mortality, with each SD higher score in neurocognition associated with 9% (auditory-vocal association function hazard ratio [HR], 0.91; 95% CI, 0.88-0.95) to 15% (full-scale IQ HR, 0.85; 95% CI, 0.81-0.88) lower mortality risk. Full scale and performance IQ and arithmetic skills were associated with an 11% (IQ HR, 0.91; 95% CI, 0.82-0.96) to 23% (arithmetic skills HR, 0.77; 95% CI, 0.72-0.83) lower mortality risk for children exposed to low adversity, crowded housing and poverty, and/or family instability, but not for those exposed to severe patterns of adversities.

**Conclusions and Relevance:**

This large US cohort study found that most childhood neurocognitive functions were associated with lower risk of premature mortality through middle adulthood, although the associations of some neurocognitive functions were diminished in the context of exposure to complex patterns of adversity. Thus, developing strong neurocognitive skills may foster long-term health resilience.

## Key Points

**Question:**

Is children’s neurocognitive development associated with lower risk of all-cause mortality in adulthood after accounting for adverse childhood experiences and other early life risk factors?

**Findings:**

In this cohort study of 49 853 children followed-up through middle adulthood, 8 of the 9 neurocognitive test scores studied were associated with a lower risk of mortality, with each SD higher scores on the neurocognitive tests associated with 9% to 15% lower risk of all-cause mortality through midadulthood. The associations between 3 neurocognitive test scores, particularly performance and full-scale intelligence quotient, with premature death varied by exposure to childhood adversity.

**Meaning:**

These findings suggest that strong neurocognitive skills in early childhood are associated with long-term health, but for those exposed to multiple, co-occurring childhood adversities, neurocognitive skills alone may not be sufficient to offset the risks.

## Teaser

This cohort study evaluates the associations of child neurocognition and adverse childhood experiences with mortality into middle adulthood.

## Introduction

Since the early 2000s when the field of cognitive epidemiology emerged, over 20 studies have uncovered a phenomenon that children with higher standardized intelligence quotient (IQ) test scores tend to live longer lives.^1,2,3,4,5,6,7^ These studies investigated the life expectancies of children born between the 1920s and 1970s^3,8,9^ following assessments of IQ at age 10 to 12 years^9,10,11^ or 18 to 20 years.^12,13,14^ In a meta-analysis, Calvin et al^15^ synthesized results from 16 cohort studies and found that each SD advantage in childhood IQ was associated with a 21% lower risk of all-cause mortality, and each SD advantage in young adulthood IQ was associated with a 25% lower mortality risk. Further adjustment for childhood socioeconomic status (SES) in a subset of the studies resulted in little attenuation of the protective effect of cognitive performance on mortality. Subsequent studies^3,9,11,16,17^ corroborated these findings.

Overall, the weight of the evidence is that cognitive abilities established in childhood, adolescence, and young adulthood provide long-term benefits for health, but several important questions remain.^18^ Less is known about the association between other aspects of neurocognitive development (eg, visual-motor function and academic skills)^19,20^ and long-term mortality risk. In addition, few studies have thoroughly examined the role of early environmental influence in the excess mortality risk attributable to childhood IQ. It is unclear if the associations between children’s neurocognitive functions and adult mortality remain after taking into account adverse childhood experiences and perinatal factors that contribute to both child development and long-term health.^21,22^ Finally, lower family SES and other childhood adversities might create a high-risk environment that weakens the protective effects of intellectual ability on premature mortality.^9,14^ It is therefore also important to clarify the extent to which the effects of neurocognitive functions on long-term health depend on the adversities that children experienced. Understanding how neurocognitive and contextual factors in early life jointly influence premature mortality through adulthood might inform early interventions to promote long-term health.

Accordingly, we investigated the associations between a range of neurocognitive functions in childhood—including visual-motor function, sensory-motor function, auditory-vocal association, IQ, and academic skills—and risk of premature mortality among children born to participants in a large prenatal cohort. We aimed to address 2 major questions: (1) whether different aspects of early neurocognitive development are associated with mortality risk decades later, after accounting for adverse childhood experiences that better captured children’s social and environmental contexts than SES alone; and (2) whether the association between neurocognitive functions and lower risk of mortality would be attenuated in the context of exposure to childhood adversity.

## Methods

### Sample

The Collaborative Perinatal Project (CPP) recruited 58 760 pregnant women between 1959 and 1966 and conducted child follow-up assessments through 1974. Informed consent was obtained from the participants. The CPP collected detailed information on the socioeconomic and racial/ethnic characteristics of participants in order to compare the CPP sample to the broader US population at the time the study was conducted.^23^ Developmental assessments of CPP offspring were conducted between 1966 and 1973 when children were aged about 7 years (mean [SD], 7.14 [0.63]). The analytic sample of this study included CPP offspring who were known to be alive by the visit at age 7 years. The current study was reviewed and approved by the institutional review board of the National Institutes of Health and followed the Strengthening the Reporting of Observational Studies in Epidemiology (STROBE) reporting guideline for cohort studies.

### Measures

#### All-Cause Mortality

Vital status of CPP offspring through 2016 was determined by matching the identifying characteristics of CPP children (name, date of birth, sex, state of birth, and race) to identifying characteristics on death certificates within the National Death Index (NDI) via a probabilistic linkage. The NDI was established in 1979 and thus does not capture the child deaths that occurred between the visit at age 7 years and December 31, 1978.

#### Childhood Neurocognitive Functions

Five neurocognitive tests were administered to CPP offspring at the visit at age 7 years including 3 neurofunctional tests: the Bender Gestalt Test (BGT; reverse-coded)^24^ for visual-motor function, the Tactile Finger Recognition Test (TFR)^25^ for sensory-motor function, the auditory-vocal association (AVA) test^26^ for abstract language thinking, the Wechsler Intelligence Scales Children (WISC)^27^ for intelligence (verbal, performance, and full-scale IQ), and the Wide Range Achievement Test (WRAT)^28^ for educational achievement (spelling, reading, and arithmetic skills). Continuous scores from these tests were standardized and the resulting *z* scores were used in analyses.

#### Childhood Adversity

Twelve adverse childhood experiences were measured. Parental harshness and neglect were based on psychologists’ observation of maternal behaviors during the visit at age 8 month. The remaining experiences assessed at the age 7 years visit either cover the period from birth to the visit at age 7 (maternal report of treatment for mental illness in the family, 2 or more changes in marital status, parent or sibling death, foster care placement, multiple residential changes, and income decline during childhood) or at age 7 (parental divorce or separation, crowded housing, welfare received, and household income below the poverty threshold).

We previously conducted a latent class analysis of these 12 adversities and identified 6 patterns of exposure^21^ (see eFigure 1 in Supplement 1): (1) low adversity (49% of the sample, low probability of experiencing any adversity; the reference group); (2) family instability (7%, relatively high probabilities of experiencing marital and residential changes); (3) crowded housing and poverty (20%, relatively high probabilities of experiencing crowded housing conditions and poverty); (4) parental harshness and neglect (3%, high probabilities of experiencing parental physical and/or emotional harshness and neglect); (5) parental separation and poverty (18%, high probabilities of experiencing parental divorce or separation, poverty, and welfare use); and (6) family loss, instability, and poverty (3%, high probabilities of experiencing the death of a parent or sibling, foster care placement, and also relatively high probability of experiencing poverty and welfare use). The first 3 groups of children either did not experience any adversity or experienced a single type of adversity (ie, either family instability or economic disadvantage only), while the other 3 groups generally experienced a constellation of 4 or more different adversities.

Potential confounders were identified based on a directed acyclic graph (DAG) developed to represent our assumptions about the causal relationships among key variables (see eFigure 2 in Supplement 1). The sociodemographic variables included child sex (male or female [referent]), mother’s self-reported race (Black, other [Asian or Hispanic], or White [referent]), mother’s age (≤20, 21-29 [referent], or ≥30), lower parental education (≤12 years or >12 years [referent]), occupation (manual occupation or unemployed or nonmanual occupation [referent]), family income below the US poverty threshold, and CPP study site (referent was largest Boston site). Maternal health included body mass index 25 or higher (calculated as weight in kilograms divided by height in meters squared), cigarette smoking during pregnancy, and self-reported treatment for mental illness during or before pregnancy. Birth outcomes included low birth weight (≤2500g), preterm birth, low Apgar scores (≤7), and any suspect or definite neurological anomaly in infancy.

### Statistical Analysis

We imputed missing data for the neurocognitive test scores and childhood adversity (missing rates ranging from 21% to 25%), as well as for the confounding variables (missing rates ranging from 0% to 9%), generating 20 complete datasets in Mplus version 8.10 (Muthén and Muthén). We plotted Kaplan-Meier curves to visualize survival probability over time for children with different levels of neurocognitive functions in R version 4.4.0 (R Project for Statistical Computing). Cox regression models of time to death or censoring were conducted in Mplus to examine how neurocognitive functions and childhood adversity were associated with the risk of all-cause mortality. Survival analyses that included childhood adversity simultaneously estimated the latent class solution for adversity and hazard ratios for the time to event outcome. Survival time was calculated as the number of years elapsed between January 1, 1979, and the date of death or censoring on December 31, 2016. Children’s age in 1979 was included in all survival analyses.

We first fitted survival models to examine the unadjusted associations of neurocognition, childhood adversity, and prenatal and neonatal confounding factors with all-cause mortality. We then fitted 9 survival models, 1 for each neurocognitive score, to investigate the association of that neurocognitive function on mortality adjusting for patterns of childhood adversity and covariates (eg, model 1 examined visual-motor function, patterns of childhood adversity, and covariates). Finally, we investigated whether the protective association between neurocognitive functions and mortality risk held for all children irrespective of exposure to childhood adversity. This was done by estimating hazard ratios (HRs) for the associations between the 9 neurocognitive functions and mortality separately according to the 6 patterns of childhood adversity and testing the interaction between each neurocognitive score and childhood adversity using Wald χ^2^ tests. A 2-sided *P *value less than .05 was considered significant. Analyses were conducted in August 2024 and June 2025.

## Results

Among the 49 853 CPP offspring, about half were male (25 226 offspring [50.6%]). A total of 23 331 (46.8%) mothers were Black, 3739 (7.5%) were other races, and 22 783 (45.7%) were White (Table 1). More than half of the parents (31 657 participants [63.5%]) were manual workers or unemployed and 22 583 parents (45.3%) had 12 years of education or less. At enrollment during pregnancy, 18 965 mothers (37.5%) lived in households below the poverty threshold, 11 367 (22.8%) were overweight or obese before pregnancy, 26 123 (52.4%) smoked during pregnancy, and 4088 (8.2%) had psychiatric illnesses during or before pregnancy. A small portion of CPP offspring were born with low birth weight (5135 offspring [10.3%]), prematurely (6880 offspring [13.8%]), had Apgar scores lower than 7 (1645 offspring [3.3%]), and had any definite or suspect neonatal neurological abnormality (5484 offspring [11.1%]). The CPP offspring were aged 13 to 20 years at the start of the follow-up on January 1, 1979, and were aged 51 to 58 years at the end of the follow-up period on December 31, 2016. There were 3553 deaths from adolescence to midadulthood identified in NDI, accounting for 7.1% of the overall sample.

**Table 1.** Sample Characteristics and Results of Unadjusted Survival Analyses for the Associations Between Each Early Life Factor and All-Cause Mortality by Midadulthood

Domain and variable	Participants, No. (%) (N = 49 853)	HR (95% CI)
Neurocognitive functions at age 7 y visits, mean (SD)		
Neurofunctional tests		
Bender Gestalt test (visual-motor function)	7.11 (3.70)	0.80 (0.77-0.82)
Tactile finger recognition (sensory-motor function)	9.22 (1.16)	0.93 (0.90-0.97)
Auditory-Vocal Association test (auditory-vocal function)	17.81 (3.77)	0.81 (0.78-0.83)
Wechsler Intelligence scale for children		
Verbal IQ	94.21 (14.20)	0.79 (0.76-0.82)
Performance IQ	98.52 (15.20)	0.75 (0.73-0.78)
Full-scale IQ	95.60 (14.96)	0.75 (0.73-0.78)
Wide Range Achievement test		
Spelling	95.68 (12.73)	0.76 (0.73-0.79)
Reading	98.27 (5.44)	0.75 (0.72-0.78)
Arithmetic	96.32 (11.11)	0.81 (0.78-0.83)
Patterns of childhood adversity between birth and age 7 y visits		
Parental harshness and neglect	1625 (3.3)	1.33 (1.06-1.66)
Parental separation and poverty	8731 (17.5)	1.70 (1.53-1.89)
Family instability	3655 (7.3)	1.28 (1.10-1.50)
Family loss, instability, and poverty	1505 (3.0)	1.31 (0.98-1.75)
Crowded housing and poverty	9901 (19.9)	1.70 (1.51-1.91)
Low adversity	22 436 (49.0)	1 [Reference]
Prenatal and neonatal confounding factors: sociodemographic, maternal health, birth outcomes, and neonatal development		
Male	25 226 (50.6)	1.79 (1.67-1.91)
Race		
Black	23 331 (46.8)	1.67 (1.56-1.79)
Other^a^	3739 (7.5)	1.06 (0.91-1.22)
White	22 783 (45.7)	1 [Reference]
Maternal age, y		
≤20	15 554 (31.2)	1.13 (1.05-1.22)
21-29	24 877 (49.9)	1 [Reference]
≥30	9422 (18.9)	1.12 (1.02-1.22)
Parent education ≤12 y	22 583 (45.3)	1.45 (1.36-1.55)
Manual occupation or being unemployed	31 657 (63.5)	1.63 (1.51-1.76)
Poverty during pregnancy	18 965 (37.5)	1.48 (1.38-1.58)
Maternal BMI before pregnancy ≥25^b^	11 367 (22.8)	1.22 (1.13-1.31)
Maternal smoking during pregnancy	26 123 (52.4)	1.16 (1.09-1.24)
Maternal psychiatric illness during or before pregnancy	4088 (8.2)	0.97 (0.86-1.10)
Low birth weight ≤2500 g	5135 (10.3)	1.26 (1.14-1.39)
Preterm birth	6880 (13.8)	1.26 (1.16-1.38)
Low Apgar scores	1645 (3.3)	1.33 (1.13-1.57)
Neonatal neurological abnormality	5484 (11.1)	1.07 (0.97-1.19)
Study site		
Buffalo, New York	2270 (4.6)	0.71 (0.55-0.91)
New Orleans, Louisiana, Charity Hospital	1775 (3.6)	2.14 (1.81-2.53)
New York, New Yorl, Columbia-Presbyterian	1994 (4.0)	1.28 (1.07-1.55)
Baltimore, Maryland, Johns Hopkins Hospital	3727 (7.5)	2.49 (2.20-2.82)
Richmond, Virginia, Medical College of Virginia	3061 (6.1)	1.87 (1.62-2.16)
Minneapolis, Minnesota, University of Minnesota Hospital	3073 (6.2)	0.94 (0.78-1.13)
New York, New York, New York Medical College	3921 (7.9)	1.55 (1.34-1.78)
Portland, Oregon, University of Oregon Medical School	3073 (6.2)	1.65 (1.42-1.92)
Philadelphia, Pennsylvania, Pennsylvania Hospital	9143 (18.3)	1.68 (1.50-1.87)
Providence, Rhode Island, Lying-In Hospital	3179 (6.4)	1.51 (1.29-1.76)
Memphis, Tennessee, University of Tennessee College of Medicine	3283 (6.6)	1.79 (1.55-2.07)
Boston, Massachusetts, Lying-In Hospital Children’s Medical Center	11 354 (22.8)	1 [Reference]

Abbreviations: BMI, body mass index; IQ, intelligence quotient.

^a^ Other included Asian or Hispanic.

^b^ Body mass index calculated as weight in kilograms divided by height in meters squared.

**Figure 1.**
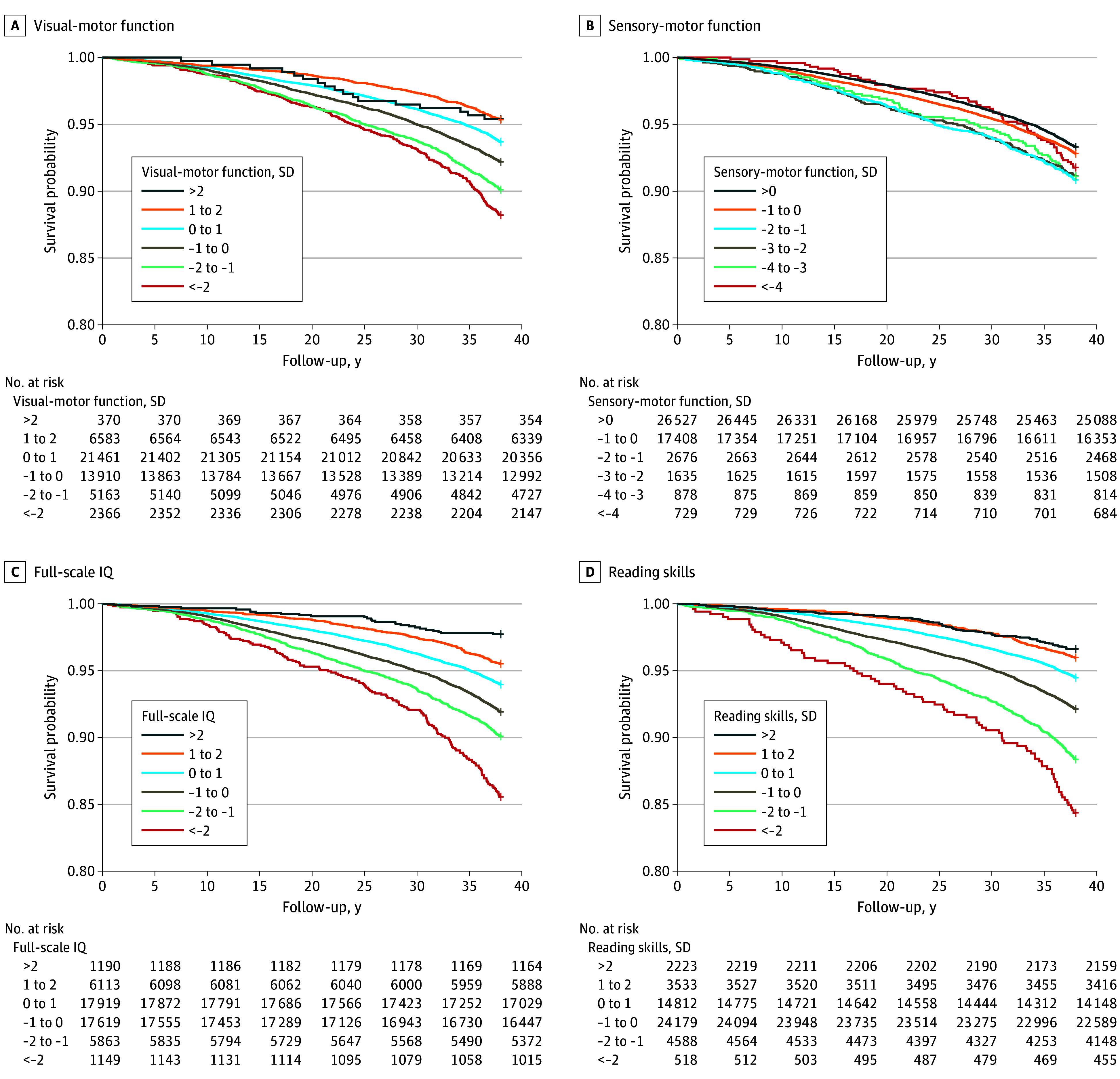
Kaplan-Meier Curves of Survival Probabilities According to Children’s Visual-Motor Functions, Sensory-Motor Function, Full-Scale Intelligence Quotient (IQ), and Reading Skills The x-axis denotes the number of years elapsed since January 1, 1979, through the follow-up period until December 31, 2016.

Kaplan-Meier curves in Figure 1 and eFigure 3 in Supplement 1 show that all 9 neurocognitive scores (eg, visual-motor function, full-scale IQ, and reading skills) were associated with an increased survival rate throughout the follow-up period, although the association was less pronounced for sensory-motor function. In unadjusted Cox models (Table 1), higher neurocognitive functions were associated with lower hazard of all-cause mortality, with estimated HRs ranging from 0.93 (95% CI, 0.90-0.97) for sensory motor function to 0.75 for full-scale and performance IQ (95% CI, 0.73-0.78) and reading skills (95% CI, 0.72-0.78). Childhood adversity was a significant factor associated with risk for all-cause mortality, with estimated HRs ranging from 1.28 for family instability (95% CI, 1.10-1.50) to 1.70 for parental separation and poverty (95% CI, 1.53-1.89) and crowded housing and poverty (95% CI, 1.51-1.91).

**Figure 2.**
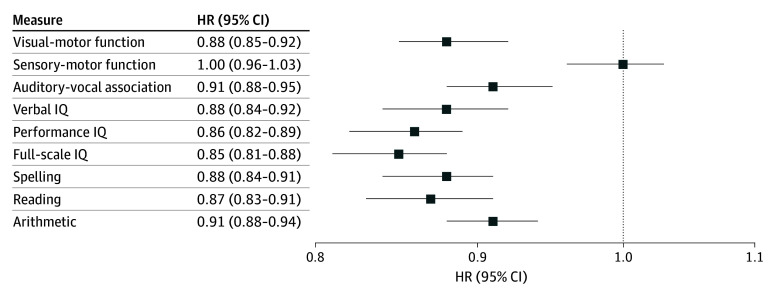
Association Between Each Neurocognitive Function and Premature Mortality After Adjusting for Childhood Adversity and Other Potential Confounding Factors The HRs were from 9 separate models. Each model inlcuded 1 neurocongitive score, the latent class variable of childhood adversity, and the whole set of prenatal and neonatal confounders. HR indicates hazard ratio; IQ, intelligence quotient.

Figure 2 and the eTable in Supplement 1 present the associations between each neurocognitive score and all-cause mortality, adjusting for childhood adversity and other potential confounders. Children’s visual-motor function (HR, 0.88; 95% CI, 0.85-0.92) and auditory-vocal association function (HR, 0.91; 95% CI, 0.88-0.95), but not sensory-motor function (HR, 1.00; 95% CI, 0.96-1.03), were associated with lower hazard of all-cause mortality. Children’s verbal IQ (HR, 0.88; 95% CI, 0.84-0.92), performance IQ (HR, 0.86; 95% CI, 0.82-0.89), and full-scale IQ (HR, 0.85; 95% CI, 0.81-0.88), were also associated with lower hazard of all-cause mortality. Similarly, children’s spelling (HR, 0.88; 95% CI, 0.84-0.91), reading (HR, 0.87; 95% CI, 0.83-0.91), and arithmetic skills (HR, 0.91; 95% CI, 0.88-0.94) were all associated with lower hazard of all-cause mortality.

The final set of analyses addressed the question of whether the protective association between neurocognitive function and mortality risk varied according to children’s pattern of exposure to adversities (Table 2). Wald χ^2^ tests suggested that the associations of 3 (ie, performance IQ, full-scale IQ, arithmetic skills) of the 9 neurocognitive scores with mortality differed according to exposure to patterns of childhood adversity. Specifically, 1 SD advantage in children’s full-scale IQ was associated with lower mortality risk among children exposed to family instability (HR, 0.81; 95% CI, 0.70-0.95), crowded housing and poverty (HR, 0.86; 95% CI, 0.77-0.96), and low adversity (HR, 0.77; 95% CI, 0.72-0.83), but not for those exposed to parental harshness and neglect; parental separation and poverty; or family loss, instability, and poverty. The class-specific associations of performance IQ and arithmetic skills with premature mortality according to childhood adversity were largely similar to those observed for full-scale IQ, with estimated HRs ranging from 0.91 (95% CI, 0.82-0.96) to 0.77 (95% CI, 0.72-0.83).

**Table 2.** Associations of Neurocognitive Test Scores With All-Cause Mortality According to Patterns of Childhood Adversity

Test	Patterns of childhood adversity, HR (95% CI)^a^						Tests of neurocognition by childhood adversity interactions	*P* value
	Parental harshness and neglect (n = 1625)	Parental separation and poverty (n = 8731)	Family instability (n = 3655)	Family loss, instability and poverty (n = 1505)	Crowded housing and poverty (n = 9901)	Low adversity (n = 24 436)		
Neurocognitive functions								
Visual-motor function	0.89 (0.72-1.10)	0.94 (0.87-1.03)	0.93 (0.80-1.07)	1.11 (0.84-1.48)	0.87 (0.80-0.95)^b^	0.83 (0.78-0.87)^b^	χ^2^_5_ = 9.58	.09
Sensory-motor function	1.00 (0.83-1.21)	0.99 (0.91-1.06)	1.01 (0.86-1.19)	1.11 (0.83-1.48)	1.01 (0.93-1.10)	0.98 (0.92-1.04)	χ^2^_5_ = 0.95	.97
Auditory-vocal association	0.96 (0.77-1.19)	0.94 (0.87-1.03)	0.84 (0.71-0.98)^b^	1.06 (0.77-1.45)	0.95 (0.87-1.05)	0.87 (0.81-0.93)^b^	χ^2^_5_ = 6.32	.28
Verbal IQ	0.88 (0.69-1.11)	0.95 (0.87-1.05)	0.81 (0.70-0.94)^b^	1.05 (0.78-1.41)	0.88 (0.79-0.98)^b^	0.84 (0.78-0.90)^b^	χ^2^_5_ = 7.63	.18
Performance IQ	0.91 (0.71-1.17)	0.97 (0.89-1.07)	0.87 (0.75-1.02)	0.89 (0.69-1.13)	0.88 (0.80-0.98)^b^	0.77 (0.72-0.83)^b^	χ^2^_5_ = 17.29	.004
Full-scale IQ	0.88 (0.68-1.12)	0.96 (0.87-1.05)	0.81 (0.70-0.95)^b^	0.95 (0.72-1.25)	0.86 (0.77-0.96)^b^	0.77 (0.72-0.83)^b^	χ^2^_5_ = 14.55	.01
Spelling	0.94 (0.75-1.17)	0.97 (0.89-1.05)	0.82 (0.71-0.95)^b^	0.98 (0.77-1.24)	0.84 (0.75-0.93)^b^	0.85 (0.79-0.92)^b^	χ^2^_5_ = 8.13	.15
Reading	0.89 (0.70-1.14)	0.93 (0.84-1.02)	0.83 (0.71-0.97)^a^	1.00 (0.77-1.29)	0.85 (0.76-0.96)^b^	0.84 (0.78-0.91)^b^	χ^2^_5_ = 4.02	.55
Arithmetic	0.95 (0.80-1.12)	1.00 (0.93-1.07)	0.86 (0.76-0.97)^b^	0.97 (0.79-1.19)	0.89 (0.82-0.96)^b^	0.85 (0.80-0.91)^b^	χ^2^_5_ = 11.10	.049

Abbreviations: HR, hazard ratio; IQ, intelligence quotient.

^a^ HRs and corresponding 95% CIs associated with 1 SD advantage in each of the neurocognitive functions for children exposed to different patterns of childhood adversity. All analyses controlled for prenatal and neonatal confounding factors.

^b^ Indicates HRs were statistically significant (ie, 95% CIs did not include 1).

## Discussion

In a large, diverse US pregnancy cohort, we examined the associations between children’s neurocognitive development and risk of mortality through middle adulthood, adjusting for adverse childhood experiences and other early life risk factors.^21,22,29,30,31,32^ For 8 of the 9 neurocognitive functions examined, higher scores were significantly associated with lower risk of mortality. This provides robust evidence that neurocognitive development across a range of areas is beneficial for adult longevity. However, we also found that for 3 of the neurocognitive functions examined (performance IQ, full-scale IQ, and arithmetic skills), this benefit does not extend to all contexts: it was attenuated in the context of exposure to patterns of adversities characterized by family disruption combined with poverty.

Consistent with prior studies,^3,4,11,15,19,20^ we found protective associations of childhood IQ and other neurocognitive functions with premature mortality. Developing strong neurocognitive skills is likely beneficial for long-term health resilience.^33^ This resilience may be operative during childhood—for many children, contemporaneously with exposure to adversity—and may also extend through adolescence and adulthood. Recent reviews highlight that many factors that promote resilience against adversity are rooted in neurocognitive strengths^34^ and can be significantly improved through interventions.^35^ Children with higher neurocognitive functions may be more likely to obtain higher educational attainment, occupation status, and income^6,36^ and to engage in more health-promoting behaviors such as less smoking, more healthful dietary choices, and more physical activity^37^ throughout adulthood. Evidence that adjusting for adult SES and adult health-related behaviors largely attenuates the effects of childhood IQ on mortality^13,15,38^ support these as potential mechanisms. According to the system-integrity hypothesis, higher mental ability may reflect overall efficiency of the brain and other bodily systems^39^ including cardiovascular, digestive, and immune systems. Individuals with greater system integrity may more adaptively respond to environmental stressors and be more resilient to disease and age-related declines, thereby increasing their likelihood of survival.^38,39^ These social, behavioral, and biological mechanisms are likely interconnected and may operate simultaneously,^1,3^ contributing to longer life expectancy by improving health indicators such as blood pressure, cardiorespiratory fitness, and chronic inflammation, thereby mitigating the risk of developing heart disease, stroke, cancer, dementia, diabetes, major depression, and schizophrenia,^18,40,41,42^ many of which are the leading causes of death.

Our exploration of interactions between neurocognition and patterns of childhood adversity revealed that the associations of certain neurocognitive functions with premature mortality varied by experiences of childhood adversity. While most neurocognitive functions examined were associated with all-cause mortality irrespective of exposure to childhood adversity,^4,9^ performance IQ, full-scale IQ, and arithmetic skills—mainly reflecting nonverbal cognitive abilities such as abstract reasoning, visual-spatial processing, and attention to detail—were beneficial for children exposed to low adversity or exposed to limited types of childhood adversity (eg, economic difficulty only). Similarly, Kajantie et al^14^ found that the association between intellectual abilities and mortality was greater among men from middle-class families compared with those from manual worker families, and Jokela et al^43^ found that higher IQ measured at ages 16 to 23 years conferred lower mortality risk only among individuals whose parents completed high school. Relatively favorable social and economic conditions might promote adaptability to the environment and improve long-term survival. In contrast, exposure to severe adversities, such as the combined challenges of family dysfunction and economic hardships, may profoundly impact children’s neurocognitive developmental trajectories^21^ and create high-risk environments in both childhood and adulthood that weaken otherwise protective effects of higher neurocognition on long-term health.

### Limitations

Although we adjusted for a broad range of confounders assessed prenatally and in early childhood, confounding due to unmeasured factors limits causal inference about the associations between neurocognitive development and premature mortality and variation according to childhood adversity. More broadly, as Iveson et al^4^ noted, any research sample is shaped by the specific time and place in which the participants lived, and this applies to our study as well. The CPP participants, born between 1959 and 1966, experienced different social, economic, educational, and health care conditions than European cohorts who lived through World War II^14,44^ or contemporaneous cohorts of children affected by events such as the COVID-19 pandemic. It is reassuring that the associations between childhood cognitive abilities and risk of mortality have generally been replicated across historical and environmental circumstances. The archival nature of the cohort gives rise to additional methodological limitations. The NDI was not established until 1979, several years after the CPP study ended; as a result, there were likely some unobserved child deaths of CPP offspring (we projected 225 such deaths based on historical mortality rates published by the US Centers for Disease Control and Prevention) and thus a left truncation bias in the survival analysis. Moreover, ascertainment of vital status was based on a probabilistic linkage to the NDI, introducing possible misclassification biases.^45^ The diversity of the CPP cohort is a major strength of the study, yet the neurocognitive tests used by the CPP may not be equally valid for all racial and ethnic groups.^46^ Controlling race, SES, and geographic context in our analyses may partially mitigate the potential test biases. While the CPP administered comprehensive neuropsychological assessments, the test for sensory-motor function only captures tactile sensation and finger movement. Further research is warranted to explore the roles of other sensory modalities (eg, temperature and pain) and their integration with complex motor functions in association with long-term health outcomes. Additionally, sample size within specific categories is always a limitation when it comes to testing interactions, even in studies having a large sample size such as the CPP, thus the interaction results, particularly among the patterns of adversity that were less common, should be interpreted with caution.

## Conclusions

Our overall conclusion is that positive neurocognitive development in young children is associated with lower risk of premature mortality through middle adulthood. That said, the results of our study suggest that for individuals exposed to multiple overlapping adversities, neurocognitive skills alone may not be sufficient to mitigate risks. Targeted approaches need to be identified to promote resilience in these high-risk groups. Future research could further explore how specific neurocognitive skills foster resilience in different contexts and refine intervention approaches to better match children to the approaches that would yield the largest anticipated benefit.

## Notes

Supplement 1. **eFigure 1.** Six Latent Classes Derived From 12 Adverse Childhood Experiences Measured Between Birth and Age 7 Years **eFigure 2. **Directed Acyclic Graph (DAG) Representing Our Conceptual Model **eFigure 3.** Kaplan-Meier Survival Curves of Survival Probabilities for Children With Different Neurocognitive Scores **eTable.** Association of Each Neurocognitive Score With All-Cause Mortality Adjusting for Childhood Adversity and Other Potential Confounders

Supplement 2.
Data Sharing Statement
