# Supplementary material for: Adverse Childhood Experiences, Neurocognitive Functions, and Long-Term Mortality Risk
Source: JAMA Netw Open. 2025 Sep 10;8(9):e2531283. doi: 10.1001/jamanetworkopen.2025.31283 (PMC12423876; doi:10.1001/jamanetworkopen.2025.31283)
Supplement: Supplement 1. — eFigure 1. Six Latent Classes Derived From 12 Adverse Childhood Experiences Measured Between Birth and Age 7 Years eFigure 2. Directed Acyclic Graph (DAG) Representing Our Conceptual Model eFigure 3. Kaplan-Meier Survival Curves of Survival Probabilities for Children With Different Neurocognitive Scores eTable. Association of Each Neurocognitive Score With All-Cause Mortality Adjusting for Childhood Adversity and Other Potential Confounders [file jamanetwopen-e2531283-s001.pdf]

Supplemental Online Content

Yu J, Haynie DL, Sundaram R, Gilman SE. Adverse childhood experiences, neurocognitive functions, and long-term mortality risk. *JAMA Netw Open*. 2025;8(9):e2531283. doi:10.1001/jamanetworkopen.2025.31283

eFigure 1. Six Latent Classes Derived From 12 Adverse Childhood Experiences Measured Between Birth and Age 7 Years
eFigure 2. Directed Acyclic Graph (DAG) Representing Our Conceptual Model
eFigure 3. Kaplan-Meier Survival Curves of Survival Probabilities for Children With Different Neurocognitive Scores
eTable. Association of Each Neurocognitive Score With All-Cause Mortality Adjusting for Childhood Adversity and Other Potential Confounders

This supplemental material has been provided by the authors to give readers additional information about their work.

© 2025 Yu J et al. *JAMA Network Open*.

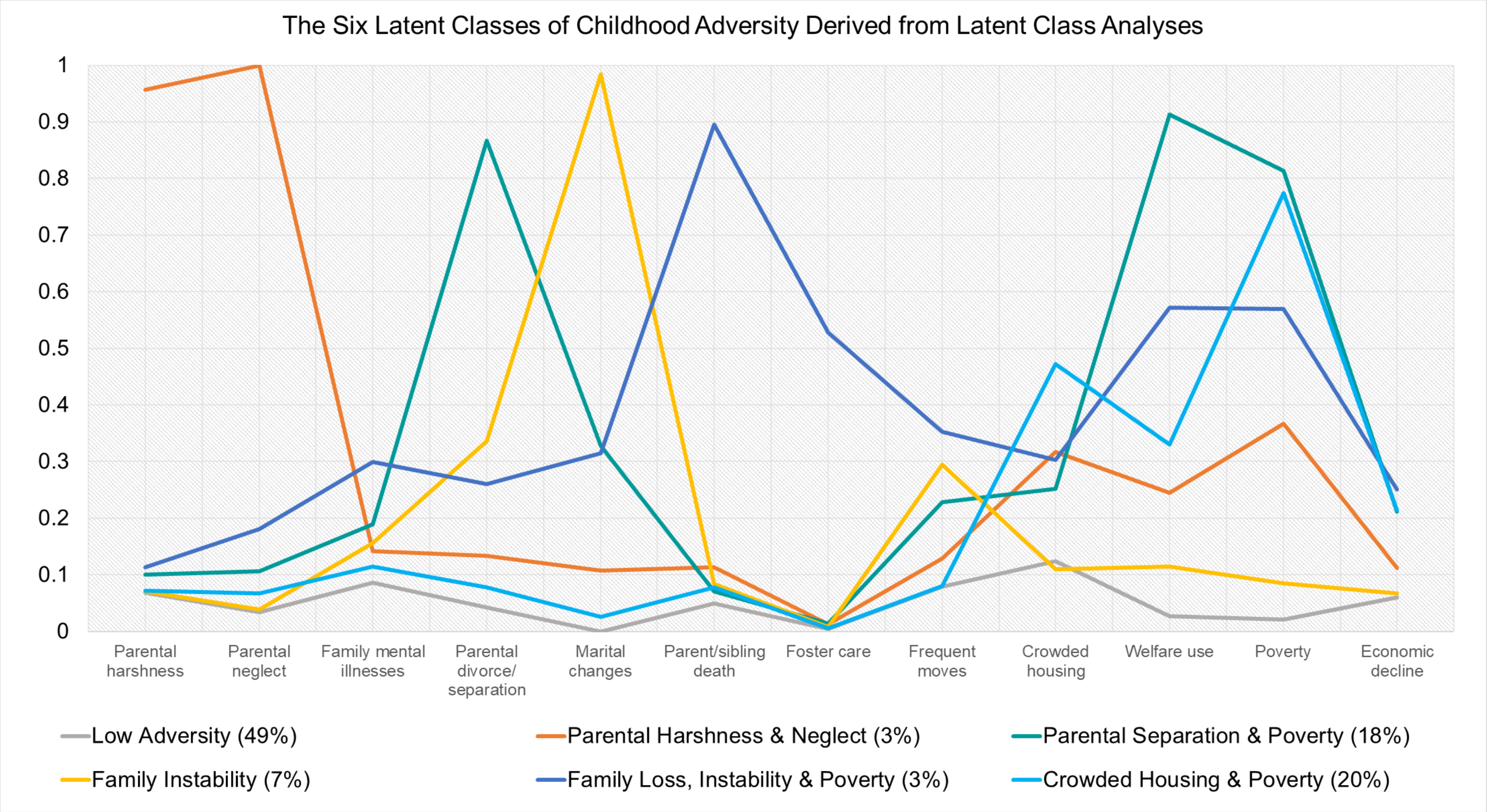

eFigure 1. Six Latent Classes Derived From 12 Adverse Childhood Experiences Measured Between Birth and Age 7 Years

Caption: The six classes: 1) Low Adversity (49% of the sample, characterized by low probability of experiencing any adversity; the reference group), 2) Family Instability (7%, characterized by relatively high probabilities of experiencing marital and residential changes), 3) Crowded Housing & Poverty (20%, characterized by relatively high probabilities of experiencing crowded housing conditions and poverty), 4) Parental Harshness & Neglect (3%, characterized by high probabilities of experiencing parental physical and/or emotional harshness and neglect), 5) Parental Separation & Poverty (18%, characterized by high probabilities of experiencing parental divorce or separation, poverty, and welfare use), and 6) Family Loss, Instability & Poverty (3%, characterized by high probabilities of experiencing the death of a parent or sibling, foster care placement, and also relatively high probability of experiencing poverty and welfare use). The figure is reprinted from the following paper: Yu J, Haynie DL, Gilman SE. Patterns of Adverse Childhood Experiences and Neurocognitive Development. *JAMA Pediatr.* 2024;178(7):678–687. doi:10.1001/jamapediatrics.2024.1318

© 2025 Yu J et al. *JAMA Network Open*.

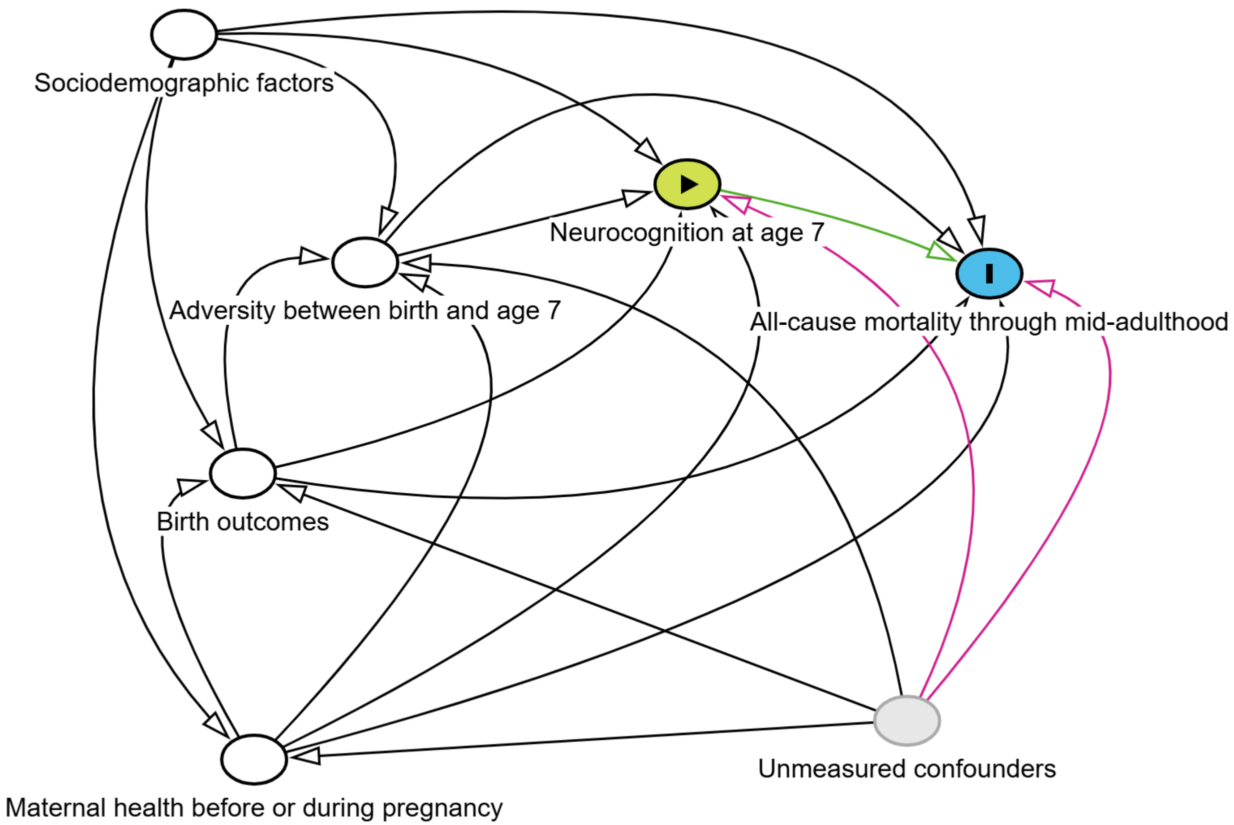

eFigure 2. Directed Acyclic Graph (DAG) Representing Our Conceptual Model (https://dagitty.net/mbPwgoaet).

Sociodemographic factors: mother race, mother age, child sex, parent education, parent occupation, living in poverty during pregnancy, and study sites reflecting geographic contexts.

Maternal health before or during pregnancy: maternal BMI before pregnancy, smoking during pregnancy, psychiatric illness before or during pregnancy.

Birth outcomes: preterm birth, low birth weight, low Apgar scores, suspect or definite neonatal neurological abnormality.

Unmeasured confounders: Some unmeasured genetic and environmental factors are likely to influence both neurocognition and risk of all-cause mortality. Many of their effects may operate through maternal health, birth outcomes, family SES, and childhood adversity, that is, factors that were accounted for in this study. Therefore, we believe that a considerable portion of the confounding from unmeasured variables has been addressed. However, we cannot rule out the possibility that some of the unmeasured confounders exert direct effects on neurocognition and all-cause mortality, which could result in residual confounding (as depicted by the pink arrows).

© 2025 Yu J et al. *JAMA Network Open*.

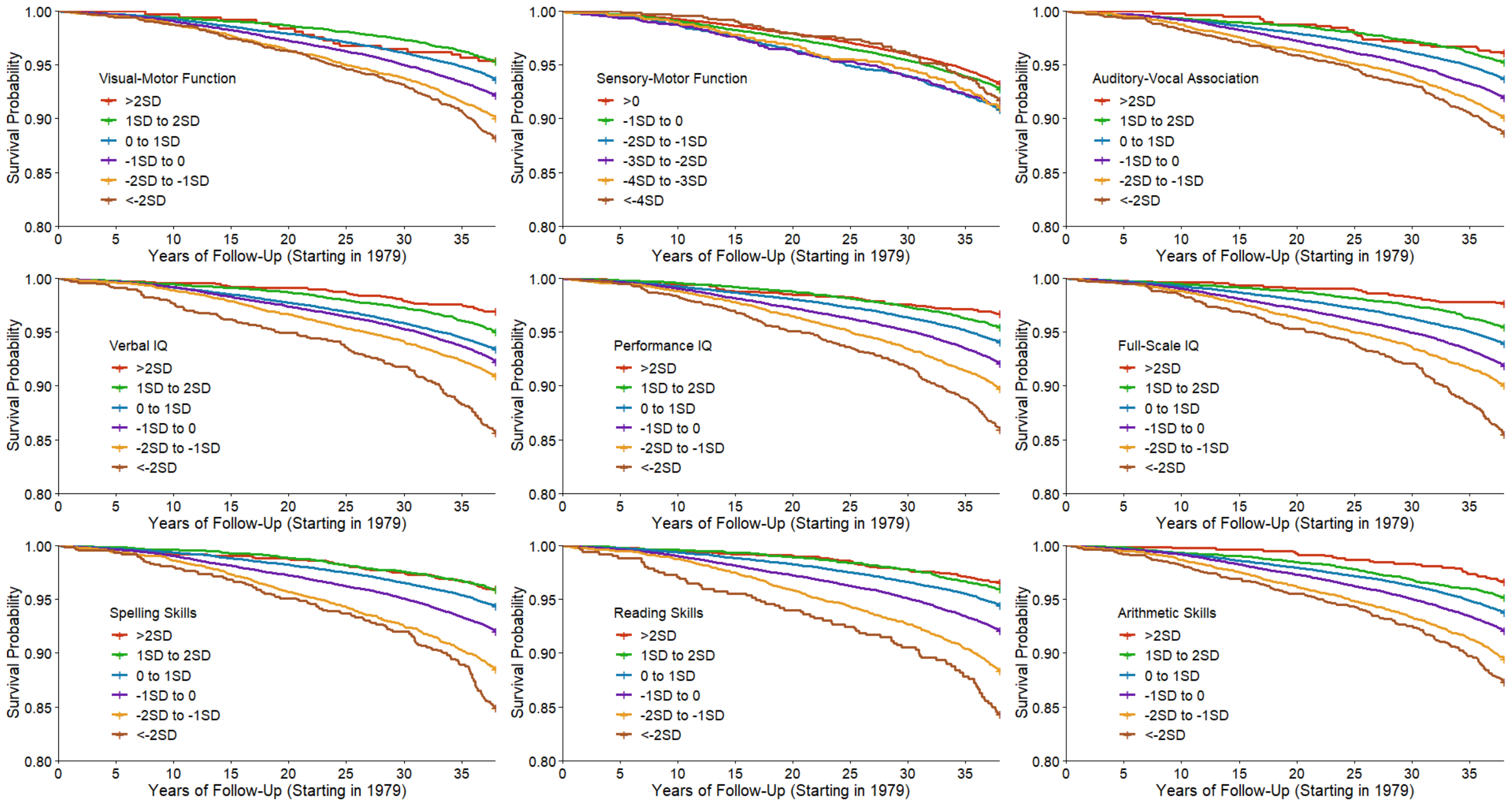

eFigure 3. Kaplan-Meier Survival Curves of Survival Probabilities for Children With Different Neurocognitive Scores

Caption: Three neurocognitive domains are neurofunctional scores (first row), IQ scores (second row), and academic skills (third row). The x-axis denotes the number of years elapsed since January 1, 1979, through the follow-up period until December 31, 2016. The y-axis indicates the cumulative probability of survival over time. The lines within each plot correspond to groups of children with different levels of standardized neurocognitive scores.

© 2025 Yu J et al. *JAMA Network Open*.

eTable. Association of Each Neurocognitive Score With All-Cause Mortality Adjusting for Childhood Adversity and Other Potential Confounders

Table 2a. Neurofunctional scores with mortality		
Model 1		**HR (95% CI)**
**Visual-motor function**		0.88 (0.85, 0.92)
**Patterns of childhood adversity** (ref = *Low Adversity*)	Parental Harshness & Neglect	1.07 (0.85, 1.35)
	Parental Separation & Poverty	1.23 (1.10, 1.39)
	Family Instability	1.17 (1.00, 1.37)
	Family Loss, Instability & Poverty	0.99 (0.73, 1.33)
	Crowded Housing & Poverty	1.21 (1.07, 1.38)
Model 2		**HR (95% CI)**
**Sensory-motor function**		1.00 (0.96, 1.03)
**Patterns of childhood adversity** (ref = *Low Adversity*)	Parental Harshness & Neglect	1.12 (0.89, 1.40)
	Parental Separation & Poverty	1.27 (1.13, 1.43)
	Family Instability	1.17 (1.00, 1.38)
	Family Loss, Instability & Poverty	1.04 (0.77, 1.40)
	Crowded Housing & Poverty	1.25 (1.10, 1.42)
Model 3		**HR (95% CI)**
**Auditory-vocal association**		0.91 (0.88, 0.95)
**Patterns of childhood adversity** (ref = *Low Adversity*)	Parental Harshness & Neglect	1.08 (0.86, 1.36)
	Parental Separation & Poverty	1.24 (1.11, 1.40)
	Family Instability	1.18 (1.01, 1.38)
	Family Loss, Instability & Poverty	1.01 (0.75, 1.36)
	Crowded Housing & Poverty	1.22 (1.07, 1.38)
**Table 2b. IQ scores with mortality**		
Model 4		**HR (95% CI)**
**Verbal IQ scores**		0.88 (0.84, 0.92)
**Patterns of childhood adversity** (ref = *Low Adversity*)	Parental Harshness & Neglect	1.07 (0.85, 1.34)
	Parental Separation & Poverty	1.22 (1.08, 1.37)
	Family Instability	1.17 (1.00, 1.37)
	Family Loss, Instability & Poverty	0.98 (0.72, 1.33)
	Crowded Housing & Poverty	1.20 (1.05, 1.37)
Model 5		**HR (95% CI)**
**Performance IQ scores**		0.86 (0.82, 0.89)
**Patterns of childhood adversity** (ref = *Low Adversity*)	Parental Harshness & Neglect	1.06 (0.84, 1.33)
	Parental Separation & Poverty	1.21 (1.08, 1.36)
	Family Instability	1.17 (1.00, 1.38)
	Family Loss, Instability & Poverty	0.97 (0.72, 1.32)
	Crowded Housing & Poverty	1.20 (1.06, 1.36)
Model 6		**HR (95% CI)**
**Full-scale IQ scores**		0.85 (0.81, 0.88)

© 2025 Yu J et al. *JAMA Network Open*.

**Patterns of childhood adversity** (ref = *Low Adversity*)	Parental Harshness & Neglect	1.05 (0.83, 1.32)
	Parental Separation & Poverty	1.20 (1.07, 1.35)
	Family Instability	1.17 (1.00, 1.38)
	Family Loss, Instability & Poverty	0.96 (0.71, 1.30)
	Crowded Housing & Poverty	1.19 (1.04, 1.35)

**Table 2c. Academic skills with mortality**		
Model 7		**HR (95% CI)**
**Spelling scores**		0.88 (0.84, 0.91)
**Patterns of childhood adversity** (ref = *Low Adversity*)	Parental Harshness & Neglect	1.07 (0.85, 1.35)
	Parental Separation & Poverty	1.21 (1.08, 1.36)
	Family Instability	1.15 (0.98, 1.35)
	Family Loss, Instability & Poverty	0.98 (0.73, 1.32)
	Crowded Housing & Poverty	1.22 (1.07, 1.38)
Model 8		**HR (95% CI)**
**Reading scores**		0.87 (0.83, 0.91)
**Patterns of childhood adversity** (ref = *Low Adversity*)	Parental Harshness & Neglect	1.07 (0.85, 1.34)
	Parental Separation & Poverty	1.21 (1.08, 1.36)
	Family Instability	1.16 (0.99, 1.36)
	Family Loss, Instability & Poverty	0.99 (0.73, 1.33)
	Crowded Housing & Poverty	1.21 (1.06, 1.38)
Model 9		**HR (95% CI)**
**Arithmetic scores**		0.91 (0.88, 0.94)
**Patterns of childhood adversity** (ref = *Low Adversity*)	Parental Harshness & Neglect	1.06 (0.85, 1.34)
	Parental Separation & Poverty	1.22 (1.08, 1.37)
	Family Instability	1.16 (0.99, 1.36)
	Family Loss, Instability & Poverty	0.97 (0.72, 1.32)
	Crowded Housing & Poverty	1.22 (1.07, 1.38)

Note: Each model included one neurocognitive score, the latent class variable of childhood adversity, and the prenatal and neonatal confounding factors. Only results for neurocognition and childhood adversity from each model are presented.

© 2025 Yu J et al. *JAMA Network Open*.
