# Supplementary material for: Adverse Childhood Experiences, Neurocognitive Functions, and Long-Term Mortality Risk
Source: JAMA Netw Open. 2025 Sep 10;8(9):e2531283. doi: 10.1001/jamanetworkopen.2025.31283 (PMC12423876; doi:10.1001/jamanetworkopen.2025.31283)
Supplement: Supplement 2. — Data Sharing Statement [file jamanetwopen-e2531283-s002.pdf]

Data Sharing Statement

Yu. Adverse Childhood Experiences, Neurocognitive Functions, and Long-Term Mortality Risk. *JAMA Netw Open*. Published September 10, 2025. doi:10.1001/jamanetworkopen.2025.31283

Data
Data available: Yes
Data types: Deidentified participant data
How to access data: The CPP data are publicly available at https://www.archives.gov/research/electronic-records/nih.html. However, the NDI data are not publicly available.
When available: With publication

Supporting Documents
Document types: None

Additional Information
Who can access the data: Researchers who are interested in the data.
Types of analyses: For research purpose.
Mechanisms of data availability: For the mortality data that are not publicly available, it may be accessed after approval of a proposal and through a signed data access agreement. Please contact stephen.gilman@nih.gov for further information.
Any additional restrictions: None
